# Urinary Analysis1Read before the annual meeting of the Pennsylvania State Veterinary Medical Association, March, 1899.

**Published:** 1899-04

**Authors:** George Jobson

**Affiliations:** Chicago, Ill.


### URINARY ANALYSIS.[1]

By George Jobson, V.S.,
CHICAGO, ILL.

My apology for not presenting a more lengthy paper to the members of this association is a lack of time to prepare one. I will, however, contribute my mite by giving some reliable qualitative tests for some of the most important pathological urinary constituents. I think that urinary analysis should play as important a part in the diagnosis of animal diseases as in those of the human family, and believe that it would help to clear up the pathology of some diseases which at present are obscure to us if more generally adopted by the members of the veterinary profession.

### UREA.

*Test No.* 1. Take one ounce of urine and concentrate by boiling to a small bulk, add strong nitric acid, and set aside to cool. Crystals of urea nitrate will form, and can be seen by the aid of the microscope. They are actahedra lozenge-shaped tablets, or hexagons.

*Test No.* 2. May be used quantitatively and qualitatively. It depends on the power of a solution of sodium hypobromite to evolve nitrogen from urea, and the gas collects in the closed end of the Doremus ureameter and displaces the solution. The tube being

[1] Read before the annual meeting of the Pennsylvania State Veterinary Medical Association, March, 1899.

marked in percentages, the amount of nitrogen gas registered represents the percentage of urea present in the urine. Normally horse's urine contains 1.5 to 2.5 per cent.; but this may vary slightly, according to the concentration of the urine. In dogs it may be 10 per cent.

To make the solution of sodium hypobromite, keep on hand a 20 per cent. solution of sodium hydrate. Fill the ureameter with this solution and add fifteen drops (1 c.c.) of bromine.

The test is made by drawing up 1 c.c. of urine into the pipette, which is marked. Insert the pipette carefully and press out the urine slowly. The following reaction occurs:

$$\underset{\text{(Urea.)}}{CoN_2H_4} + \underset{\text{(Sodium hypobrom.)}}{3Na\ Bro} \quad \underset{\text{(Carbonic acid.)}}{Co_2{}''} + \underset{\text{(Nitrogen.)}}{N_2} + \underset{\text{(Water.)}}{2H_2O} + \underset{\text{(Sod. Brom.)}}{3\ Na\ Br.}$$

### ALBUMIN.

*Test No.* 1. Acidulate a quantity of urine with a few drops of acetic acid, then add a saturated solution of potassium ferrocyanide. This gives a precipitate when much albumin is present, and produces an opalescence when there is only a trace. This test will show as little albumin, as 1 part in 60,000.

*Test No.* 2. Pour a small quantity of fuming nitric acid into a test-tube and add a little warm urine in such manner that the urine floats on top. If albumin is present a white zone is formed at the line of demarcation. If the urine is not warmed before making the test, urates may give a similar reaction.

A great many substances beside albumin will react to any test which is used for this substance, as salol, or also resins, in the urine. But by an addition of an excess of potassium ferrocyanide these precipitates are redissolved. Before testing for albumin urine must be made perfectly clear by filtering, otherwise you will not be able to detect traces.

### SUGAR.

*Test No.* 1. Test Fehling's solution, which is a mixture of copper sulphate, caustic soda, and Rochelle salts, by boiling. If it remains clear it is in good condition. Take equal parts of urine and Fehling's solution and raise to the boiling-point. A yellowish or reddish precipitate stands for sugar.

*Test No.* 2. This is probably the most reliable test known for sugar. To two drachms of urine add the point of a knifeful of phenylhydrazin chloride, and the same amount of sodium acetate crystals, shake thoroughly until dissolved. Then place in a hot water-bath just below the boiling-point for thirty minutes. Remove the test-tube, cool the solution under running cold water, and examine some of the yellow precipitation with a microscope. If sugar be present yellow needle-like crystals are seen.

Before testing for sugar, if albumin is present, remove by acidulating with acetic acid, boil, and filter.

### BLOOD.

To a little tr. guaiac add an excess of peroxide of hydrogen. To this add the suspected urine. A blue color stands for blood.

### PUS.

Acidulate a small portion of urine with acetic acid; filter through a double filter; then treat the moist residue on the filter-paper with a few drops of tr. guaiac, which gives a blue tinge.

### PHOSPHATES.

Phosphates are usually found in the urine as an amorphous white substance, which clears up on addition of acetic acid. Very often neutral or alkaline urine will become cloudy when boiled. This may be due to albumin or phosphates. They may be distinguished by the acetic-acid test, which dissolves phosphates but not albumin.

